# Effects of Lidocaine and Src Inhibition on Metastasis in a Murine Model of Breast Cancer Surgery

**DOI:** 10.3390/cancers11101414

**Published:** 2019-09-22

**Authors:** Thomas P. Wall, Peter D. Crowley, Aislinn Sherwin, Andrew G. Foley, Donal J. Buggy

**Affiliations:** 1 Department of Anaesthesiology & Perioperative Medicine, Mater University Hospital, School of Medicine, University College Dublin, D07 KH4C Dublin, Ireland; aislinn.sherwin@gmail.com (A.S.); donal.buggy@ucd.ie (D.J.B.); 2 Conway Institute for Biomolecular and Biomedical Research, School of Medicine University College Dublin, D04 V1W8 Dublin, Ireland; peter.crowley@ucdconnect.ie; 3 Berand Neuropharmacology Ltd., NovaUCD, D04 V1W8 Dublin, Ireland; andrew.foley@berand.ie; 4 Outcomes Research, Cleveland Clinic, Cleveland, OH 44195, USA

**Keywords:** cancer recurrence, metastasis, anaesthesia, local anaesthetics, lidocaine, Src, bosutinib

## Abstract

Breast cancer recurs in 20% of patients following intended curative resection. In vitro data indicates that amide local anaesthetics, including lidocaine, inhibit cancer cell metastasis by inhibiting the tyrosine kinase enzyme Src. In a murine breast cancer surgery model, systemic lidocaine reduces postoperative pulmonary metastases. We investigated whether the additional administration of bosutinib (a known Src inhibitor) influences lidocaine’s observed beneficial effect in this in vivo model. Female BALB/c mice (*n* = 95) were inoculated with 25,000 4T1 cells into the mammary fad pad and after 7 days the resulting tumours were excised under sevoflurane anaesthesia. Experimental animals were randomized to one of four treatments administered intravenously prior to excision: lidocaine, bosutinib, both lidocaine and bosutinib in combination, or saline. Animals were euthanized 14 days post-surgery and lung and liver metastatic colonies were evaluated. Post-mortem serum was analysed for MMP-2 and MMP-9, pro-metastatic enzymes whose expression is influenced by the Src pathway. Lidocaine reduced lung, but not liver metastatic colonies versus sevoflurane alone (*p* = 0.041), but bosutinib alone had no metastasis-inhibiting effect. When combined with lidocaine, bosutinib reversed the anti-metastatic effect observed with lidocaine on sevoflurane anaesthesia. Only lidocaine alone reduced MMP-2 versus sevoflurane (*p* = 0.044). Both bosutinib (*p* = 0.001) and bosutinib/lidocaine combined (*p* = 0.001) reduced MMP-9 versus sevoflurane, whereas lidocaine alone did not. In a murine surgical breast cancer model, the anti-metastatic effects of lidocaine under sevoflurane anaesthesia are abolished by the Src inhibitor bosutinib, and lidocaine reduces serum MMP-2. These results suggest that lidocaine may act, at least partly, via an inhibitory effect on MMP-2 expression to reduce pulmonary metastasis, but whether this is due to an effect on Src or via another pathway remains unclear.

## 1. Introduction

Breast cancer is the leading cause of cancer-related death in women, resulting in over 620,000 deaths worldwide in 2018 [1]. Early diagnosis and excision of the primary tumour may be curative, but metastatic recurrence following surgery is common, and typically fatal [2]. Perioperative events, including anaesthesia, are hypothesised as potentially influencing development of metastatic disease [3]. It is postulated that manipulation of the primary tumour during surgery can dislodge tumour cells into the circulation which may then seed distant organs to eventually form metastases [4]. Direct or indirect effects of anaesthetic agents on such circulating tumour cells during surgery could be important in altering the risk of post-operative metastatic progression [5].

Amide local anaesthetics (ALAs) have shown promising results in pre-clinical studies examining their effects on cancer. Lidocaine is the only ALA with a safety profile consistent with intravenous use and is commonly administered as an antiarrhythmic and perioperatively both as an analgesic agent and to hasten the return of gut motility following bowel surgery [6]. In vitro evidence suggests that lidocaine has inhibitory effects on cancer cells, and is associated with reduced cancer cell viability, migration and invasion in breast, hepatocellular and lung adenocarcinoma cells [7,8,9].

In vitro evidence of potential cancer-resisting effects of lidocaine is also supported by in vivo data: lidocaine treatment improved survival in mice with peritoneal carcinomatosis, and in mice inoculated with hepatocellular cell xenografts [7,8]. We previously adapted a murine model of breast cancer to replicate surgical perioperative conditions. Reduced pulmonary metastatic colonies at 14 days post-excision of the primary tumour was observed with perioperative systemic lidocaine in addition to sevoflurane, compared with sevoflurane alone [10,11], and again when lidocaine was co-administered with cisplatin [12].

The mechanism underlying this inhibitory effect of lidocaine on metastasis is unknown. A variety of mechanisms have been postulated, including inhibition of Src [13,14,15,16]. Src is an intra-cellular, non-receptor tyrosine kinase with pleiotropic biologic effects including regulation of cell survival, migration and proliferation [17]. Over-expression of Src has been implicated in the progression of numerous human cancers including breast, colon and pancreatic cancer [18]. Src activation increases the expression of matrix metalloproteinases (MMPs), including MMP-2 and MMP-9, enzymes which degrade extracellular matrix and contribute to cancer cell migration and invasion [19,20,21,22].

We tested whether the in vivo anti-metastatic effect of lidocaine in a murine surgical model of breast cancer is influenced by an agent known to act on the Src pathway. We examined the effect of the novel Src inhibitor bosutinib alone and in conjunction with lidocaine in an in vivo setting, using a modified 4T1-BALB/c murine allograft model.

## 2. Results

### 2.1. Baseline Characteristics

**Figure 1:**
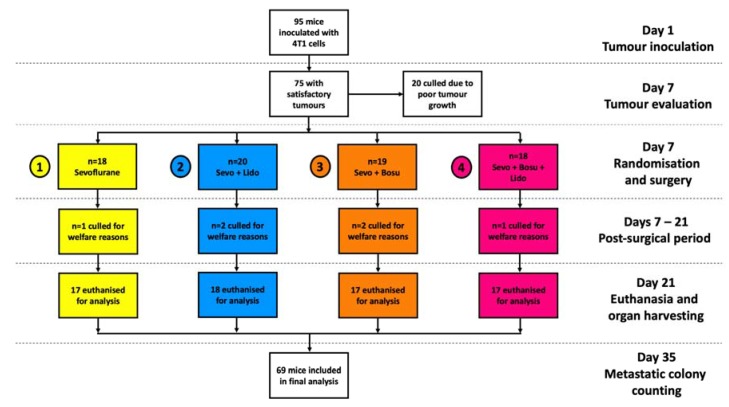
Flow diagram of in vivo experimental protocol and animal numbers. On day 1 of experiment, 4T1 breast cancer cells were inoculated into the mammary fat pad of female BALB/c mice (*n* = 95). After 7 days, mice with palpable tumours (*n* = 75) underwent tumour excision under sevoflurane anaesthesia, plus randomised perioperative drug treatment. Group 1 received sevoflurane alone. In addition to sevoflurane, Group 2 received i.v. lidocaine, Group 3 received i.v. bosutinib, and Group 4 received i.v. lidocaine plus bosutinib. On day 21, the mice were euthanized and lungs, liver and serum sampled for analysis. Lung and liver samples were cultured for 14 days with 6-thioguanine exposure and any metastatic colonies present were counted on day 35 of the experiment. Abbreviations: Sevo, sevoflurane; Bosu, bosutinib; Lido, lidocaine.

Mean animal weights and tumour sizes on the day of surgery were similar across the four groups. Weights in grams on the day of surgery were (mean ± SD): 18.8 ± 1.2, 19.0 ± 1.7, 19.5 ± 1.4 and 19.5 ± 1.0 in Groups 1–4 respectively. Calculated tumour volumes immediately prior to resection in mm^3^ were: 7.5 ± 6.6, 8.1 ± 5.0, 9.1 ± 6.8 and 8.8 ± 6.4 in Groups 1–4 respectively. Twenty animals were culled prior to randomisation due to failure of tumour growth and six animals were culled for post-surgical welfare reasons (1–2 animals per group). Sixty-nine animals remaining were included in the final analysis (Figure 1).

### 2.2. Effects on Metastasis

**Figure 2:**
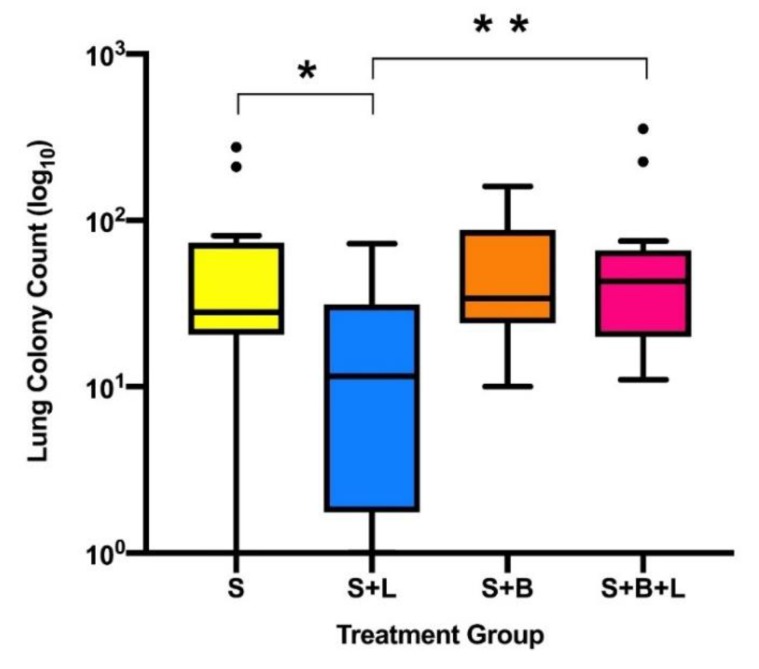
Lung metastatic colony counts by treatment group (median, IQR and range). * indicates difference between group S and S + L (*p* = 0.041), ** indicates difference between groups S + L and S + B + L (*p* = 0.011). • indicates outliers by Tukey’s method (>1.5 × IQR). Abbreviations: S, sevoflurane alone; S + L, sevoflurane and lidocaine; S + B, sevoflurane and bosutinib; S + B + L, sevoflurane, lidocaine and bosutinib.

**Figure 3:**
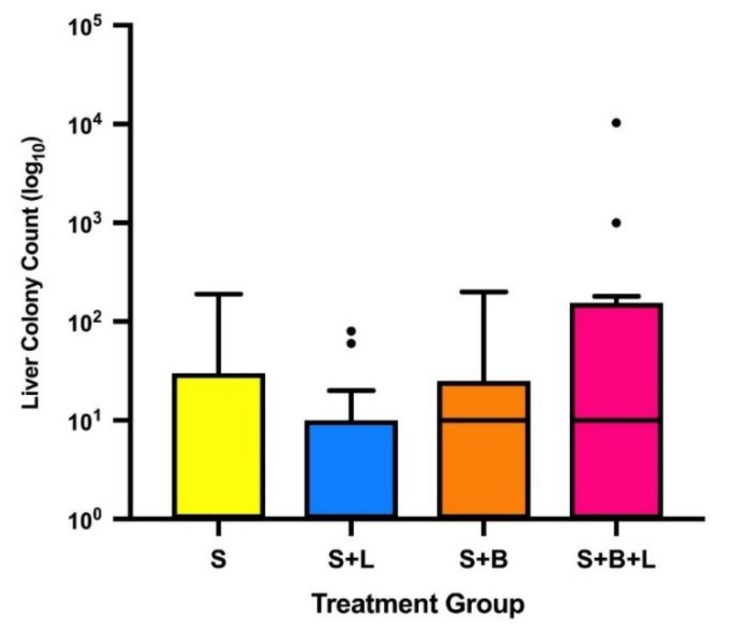
Liver metastatic colony counts by treatment group (median, IQR and range). Note that the median value of groups S and S + L is 0. • indicates outliers by Tukey’s method (>1.5 × IQR). Abbreviations: S, sevoflurane alone; S + L, sevoflurane and lidocaine; S + B, sevoflurane and bosutinib; S + B + L, sevoflurane, lidocaine and bosutinib.

In the surgical model of murine 4T1 breast cancer, i.v. lidocaine administered with sevoflurane anaesthesia during primary tumour excision significantly reduced lung metastatic colony count at 2 weeks post-operatively compared to sevoflurane anaesthesia alone. Animals that received i.v. lidocaine had reduced lung metastatic colony counts (median (IQR)): 12 (1.75–31) versus the sevoflurane control group: 28 (20.5–73.5), *p* = 0.041 (Figure 2). There was no significant difference in liver metastatic colony counts between the same two groups: 0 (0–10) versus 0 (0–30), *p* > 0.999 (Figure 3).

Lidocaine alone was the only treatment group that reduced lung metastatic colony counts compared to the sevoflurane control group. No significant difference was seen in lung colony counts between the sevoflurane control group and the two groups receiving bosutinib. Bosutinib did not reduce the lung metastatic colony count: 34 (24–87), *p* > 0.999, or the liver colony count: 10 (0–25), *p* > 0.999, when compared to sevoflurane alone. Lidocaine and bosutinib in combination compared to bosutinib alone resulted in no significant difference in metastatic colony counts in either lung or liver (*p* > 0.999 for both). Lidocaine treatment alone resulted in reduced lung colonies compared to lidocaine and bosutinib in combination (*p* = 0.011). No treatment significantly reduced liver colony count compared to the sevoflurane group. There was no significant difference in primary tumour recurrence between the treatment groups: 3, 3, 4 and 3 tumours recurred in Treatment Groups 1–4, respectively.

### 2.3. Serum MMP-2 and MMP-9

**Figure 4:**
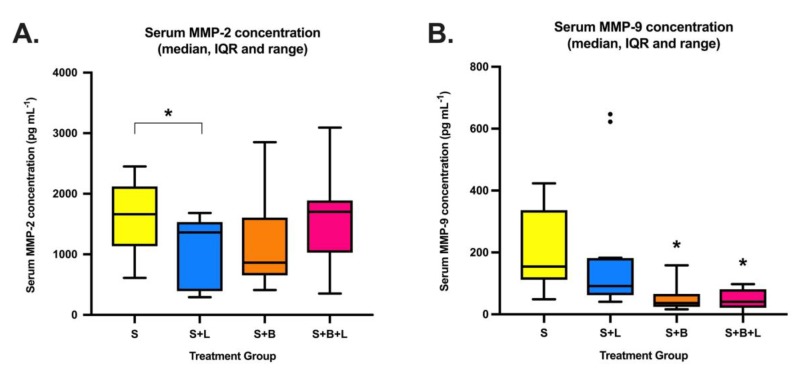
Serum MMP-2 (**A**) and MMP-9 (**B**) concentrations by treatment group (median, IQR and range) measured from samples at 14 days post-primary tumour excision (*n* = 12 animals per group). * indicates significant difference compared to group S (*p* < 0.05). • indicates outliers by Tukey’s method (>1.5 × IQR). Abbreviations: S, sevoflurane alone; S + L, sevoflurane and lidocaine; S + B, sevoflurane and bosutinib; S + B + L, sevoflurane, lidocaine and bosutinib.

Lidocaine reduced mean MMP-2 serum concentration 14 days post-tumour resection compared to sevoflurane: 1099 ± 787 pg mL^−1^ (mean ± SD) versus 1598 ± 586 pg mL^−1^, *p* = 0.044 (Figure 4). Neither bosutinib group had significantly different mean MMP-2 compared to the sevoflurane group. A significant difference was noted in serum MMP-9 concentrations between the sevoflurane group: 199.7 ± 129.5 pg mL^−1^ and the two groups that received bosutinib–those receiving bosutinib alone: 48.0 ± 23.8 pg mL^−1^, *p* = 0.001, and those receiving lidocaine/bosutinib: 47.7 ± 35.6, *p* = 0.001. Lidocaine did not significantly reduce serum MMP-9 versus sevoflurane alone (*p* = 0.874).

## 3. Discussion

In this study, we investigated whether the Src pathway is involved in the mechanism of action of the anti-metastatic effect of lidocaine in an animal model. To examine this, we used bosutinib (SKI-606)—an Src/Bcr-Abl tyrosine kinase inhibitor used to treat haematological malignancies [23]. Bosutinib has been assessed in breast cancer treatment clinical trials, with largely disappointing results [24]. Although bosutinib’s action is not entirely isolated to Src inhibition, it is more selective than similar agents such as dasatinib which also target a range of cancer-implicated enzymes such as c-KIT [25].

This study confirms the previously observed effect of perioperative i.v. lidocaine on reducing post-operative pulmonary metastasis [10,11,12]. Given the very short duration of exposure of the subject to the drug, it seems plausible that this anti-metastatic result is due to an effect by lidocaine on circulating tumour cells (CTCs) released into the circulation during the procedure itself. If a short-duration perioperative lidocaine infusion reduces pulmonary metastasis via an Src-inhibiting effect, then it is plausible to expect a similar anti-metastatic effect with a known Src inhibitor such as bosutinib. Indeed, research has shown that just a single i.v. dose of saracatinib—a similar dual Bcr—Abl/Src inhibitor to bosutinib, inhibits pulmonary metastasis when administered shortly after i.v. injection of sarcoma cells into a murine model [26]. The clinical implication of isolating a beneficial anti-metastatic effect of perioperative lidocaine to an effect on the Src pathway is clear—if lidocaine produces such an effect via Src, then other more potent Src influencing agents may produce even greater protective effects if given perioperatively, perhaps without the significant risks of toxicity that lidocaine carries.

However, perioperative bosutinib was not shown to have an anti-metastatic effect in our model. This may not be entirely surprising, given that standard treatment with bosutinib requires weeks in order to achieve a clinical effect, and as previously mentioned, in clinical settings breast cancer is not highly responsive to bosutinib [24,27]. If lidocaine does act via Src inhibition in reducing metastasis then it would be reasonable to expect that co-treatment with bosutinib would result in additive effects leading to greater reduction of lung metastasis. On the contrary, bosutinib administered with lidocaine abolished the reduction in pulmonary metastasis previously observed with lidocaine alone.

Although lidocaine inhibits Src in an inflammatory setting in vitro, the exact mechanism of this action is unclear [28]. This effect may be due to competition with adenosine triphosphate (ATP) at its binding site on Src [28]. Bosutinib inhibits Src by reversible competition at the ATP binding site [29]. It is possible that lidocaine and bosutinib compete for the ATP-binding site on Src, but bosutinib has greater affinity and binds more avidly, yet has lower potency once bound than lidocaine—thereby blocking the observed anti-metastatic effect of lidocaine.

Another potential explanation for our findings is that Src effects on cancer cells are complex, and using multiple agents that act on Src may not always lead to readily explicable results. Treatment with dasatinib, an Src inhibitor with a broader enzymatic target range than bosutinib [25], is associated with both increased lung metastases [30], and with reduced lung metastases in non-surgical 4T1/BALB-c models [31]. Interestingly, in vitro studies of human triple-negative breast cancer demonstrated strikingly differential effects of bosutinib and dasatinib [32]. Whereas bosutinib (≤1 µM) had minimal inhibitory effects on cell proliferation and viability, dasatinib showed potent inhibitory effects at much lower concentrations. Used in combination, bosutinib reduced the inhibitory effect of dasatinib and actually enhanced cancer cell proliferation. Clearly, combined use of Src inhibitors does not always lead to the additive beneficial effects that might be expected. The authors postulate that bosutinib induces kinome re-programming, activating alternative cellular signalling pathways resulting in resistance to dasatinib’s anti-proliferative effects.

As we have noted in previous studies, the beneficial effect of lidocaine does not appear to extend to liver metastasis [10,11,12]. This may be due to slower 4T1 metastatic spread to the liver—it has been shown that a period of 22 days post-inoculation are required for hepatic metastases to become detectable in 60% of animals, whereas pulmonary metastases are detectable in 100% of animals at 14 to 18 days post-inoculation [33]. Waiting for a longer period post-tumour excision before organ sampling would perhaps improve the likelihood of detecting an effect on hepatic metastases, however this would need to be balanced against the potential impact on animal welfare of the rapidly growing synchronous pulmonary metastases.

Analysis of post-mortem serum MMP-2 and MMP-9 yielded differing findings. Mean MMP-2 was reduced in the lidocaine-only group compared to the sevoflurane group, as may be expected if lidocaine acts as a Src-inhibitor and reduces downstream MMP-2 production. Similar to the lung metastasis results, this MMP-2 reduction was not induced by bosutinib, or by combined lidocaine/bosutinib—again suggesting that bosutinib blocks lidocaine’s effects. However, MMP-9 concentration was not reduced by lidocaine, but was reduced in both bosutinib groups. Together, these results suggest that lidocaine and bosutinib have differential effects on MMP expression, with MMP-9 being more sensitive to bosutinib—individual MMP expression is driven by numerous factors that lidocaine and bosutinib may influence differently, and by non-Src mechanisms [19].

Ideally, the effect of lidocaine on Src activation in circulating tumour cells (CTCs) released perioperatively would be quantified. This is technically very difficult however, as CTCs released as the primary tumour is manipulated would reach peripheral targets very rapidly and be removed from the circulation. A study examining CTCs in a murine breast cancer model found that there were only between 1 and 7 CTCs remaining per mL of blood 1 hour following an intravenous injection of 10^6^ or 2 × 10^6^ MDA-MB-231 cells in the lateral tail vein of the mice [34]. Such a rapid removal of cells from the circulation makes the timing of surgical excision and subsequent blood sampling (requiring cardiac puncture and sacrifice of the animal) to ensure capture of an adequate number of CTCs for Src evaluation extremely challenging. Ongoing refinements of CTC-based experimental techniques may render this feasible in the future.

## 4. Methods

### 4.1. Tumour Model

The BALB/c 4T1 murine breast cancer model is a commonly used in vivo syngeneic model of aggressive breast cancer, typically resulting in rapid metastatic spread similar to human disease. 4T1 cells are resistant to 6-thioguanine, enabling metastatic cells present in distant organs to be quantified using tissue culture techniques [33]. Spontaneous metastasis of tumour cells in this model occurs on average 8 days following inoculation of 4T1 tumour cells into the mammary fat pad. In this experiment, tumour excision was performed after 7 days, typically before development of metastatic disease, thereby allowing the effect of perioperative agents on subsequent metastasis to be quantified.

### 4.2. Test Animals

Ethical approval for this study (Ethical Committee ID AREC-17-25-Buggy) was granted by the University College Dublin Animal Research Ethics Committee (Chairperson Dr F. Leonard) on 8 August 2018 and authorized by the Health Products Regulatory Authority of Ireland (HPRA). The ARRIVE guidelines for optimising the reporting of animal use in research were followed [35]. Experiments were performed as per the European Union (EU) directive on animal research (EU2010/63) [36]. 8–10-week-old female BALB/c mice (Charles River Laboratories, UK) were used as the experimental animals. Mice were kept in pathogen-free conditions and housed in individually ventilated cages (5 animals per cage). The animals were maintained in a 12-h light/dark cycle at standard temperature and humidity and provided with ad libitum access to food and water.

### 4.3. Establishing the Tumour

On day 0 of the study, the mice were subcutaneously inoculated with 2.5 × 10^4^ 4T1 cells in 0.025 mL of RPMI with a 30-gauge hypodermic needle into the fat pad of the inferior right inguinal mammary gland [33]. Tumour growth was assessed daily, and the size of palpable tumours was measured using calipers. Animal welfare was monitored every day via assessment of weight, appearance, behaviour and the grimace scale [37].

### 4.4. Treatment Groups

On the day of surgery, animals were allocated by blocked stratified randomisation to one of 4 perioperative treatment groups as outlined below, each group consisting of 18–20 animals (Figure 3):

Sevoflurane anaesthesia (S): Sevoflurane 3% in 50% oxygen-air, followed by an i.v. bolus and 25-min i.v. infusion of saline (of equal volume to that received by groups 2–4).

Sevoflurane anaesthesia with intravenous lidocaine (S + L): Equivalent inhalational anaesthesia to Group 1, i.v. lidocaine 1.5 mg kg^−1^ bolus followed by a 25-min i.v. infusion of lidocaine at 2 mg kg^−1^ h^−1^.

Sevoflurane anaesthesia with intravenous bosutinib (S + B): Equivalent inhalational anaesthesia to Group 1, i.v. bosutinib bolus 5 mg kg^−1^ followed by a 25-min i.v. infusion of saline.

Sevoflurane anaesthesia with intravenous bosutinib and lidocaine (S + B + L): Equivalent inhalational anaesthesia to Group 1, i.v. bolus of bosutinib 5 mg kg^−1^ then i.v. lidocaine 1.5 mg kg^−1^ followed by a 25-min i.v. infusion of lidocaine 2 mg kg^−1^ h^−1^.

### 4.5. General Anaesthesia

At 7 days post-inoculation of tumour cells, if no palpable tumour was present the animal was excluded from the study and euthanized. Otherwise, palpable tumours were excised under general anaesthesia using sevoflurane (AbbVie, Chicago, IL, USA) delivered at a concentration of 5% in 50% oxygen into a closed induction box. After confirming loss of pedal withdrawal, anaesthesia was maintained with 3% sevoflurane in 50% oxygen via face mask. Depth of anaesthesia was deemed sufficient when loss of the righting and palpebral reflexes and absent pedal withdrawal were confirmed. Anaesthesia was discontinued after 30 min and the animal was recovered.

### 4.6. Tumour Excision

Following shaving, the surgical site was cleaned and disinfected with chlorhexidine gluconate 20 mg mL^−1^ and isopropyl alcohol 0.70 mL mL^−1^ (ChloraPrep^®^, CareFusion UK Ltd., Basingstoke, UK). A 26-gauge cannula (Hospira, Lake Forest, IL, USA) was placed in a lateral tail vein to provide intravenous access for drug administration. Standard surgical technique was used to excise the tumour. The surgical wound was closed using interrupted 6.0 polypropylene sutures (Ethicon, Bridgewater, NJ, USA). A uniform intravenous infusion duration of 25 min was maintained for every animal, following which the cannula was removed.

### 4.7. Administration of Analgesics, Lidocaine and Bosutinib

After anaesthesia was induced and prior to surgical incision, paracetamol 200 mg kg^−1^ and carprofen 10 mg kg^−1^ were given subcutaneously (SC) as pre-emptive analgesia. Intravenous lidocaine was administered as a 1.5 mg kg^−1^ bolus via the tail-vein cannula followed by an infusion of 2 mg kg^−1^ h^−1^. Animals in Treatment Groups 1 and 3 received an equivalent infusion volume of normal saline. Intravenous bosutinib was administered as a 5 mg kg^−1^ bolus via the same route. All mice received 5 post-operative SC doses of paracetamol (200 mg kg^−1^) and carprofen (10 mg kg^−1^) in warmed saline (20 mL kg^−1^) at 12-h intervals. Additional doses were given as required using the grimace scale as an indicator of pain [37].

### 4.8. Quantification of Distant Metastasis

On study day 21, each mouse was euthanized by cervical dislocation (without anaesthesia or sedation), followed by decapitation and blood collection. The lungs and liver (in their entirety) were excised using aseptic technique. Lung samples were treated with 5 mL collagenase IV in Hank’s balanced salt solution (HBSS) (~125 units mL^−1^) (Sigma-Aldrich, St. Louis, MO, USA), and liver samples were treated with collagenase I (~125 units mL^−1^) (Sigma-Aldrich) and hyaluronidase (~500 units mL^−1^) (Sigma-Aldrich) in 5 mL HBSS. Organ samples were passed through a cell strainer to isolate cells and then cultured in medium containing 60 µM 6-thioguanine (Sigma-Aldrich) at 37 °C in air with 5% CO_2_ for 14 days. At that point, the plates were fixed using 100% methanol and stained with 0.03% wv^−1^ methylene blue for 5 min. Each metastatic colony formed in this manner results from a single (or small number of) 4T1 cells. Two researchers independently inspected the plates and were blinded to the experimental treatment groups and to each other’s interpretation, and colony numbers were counted as per gross appearance [33].

### 4.9. Serum Analysis

Blood samples were collected following decapitation. All samples analysed were from animals with pulmonary metastases within each treatment arm (*n* = 12 per group). Blood samples were centrifuged and serum was collected. An amount of 10 µL of serum was diluted in PBS to a final volume of 50 µL. Samples were analysed using mouse MMP-2 and MMP-9 enzyme-linked immunosorbent assay (ELISA) kits (Abcam, Cambridge, UK) according to the manufacturer’s protocols. MMP-2 and MMP-9 concentration was calculated by measuring sample absorbance at 450 nm.

### 4.10. Statistical Analysis

The primary end point was the number of pulmonary metastatic colonies. Secondary end points were liver metastatic colonies and the presence or absence of local recurrence of breast tumour. A 50% reduction in colony count was deemed likely to be oncologically significant, therefore the study was designed to detect this level of reduction in lidocaine-treated groups, i.e., from 50 to 25 metastatic colonies. Previous experiments demonstrated standard deviation of pulmonary colony counts to be in the order of 25–45 [10,12]. Taking the middle of that range (i.e., SD = 35) in order to increase the likelihood of detecting any difference and assuming a Type I error = 0.05, and Type II error = 0.2 (80% power), then a desired sample size of approximately *n* = 20 animals per group was estimated. Use of the D’Agnostino and Pearson omnibus normality test found that the cell viability and migration and colony count data were non-normally distributed, therefore differences were analysed using the Kruskal–Wallis test with posthoc Dunn’s test to correct for multiple comparisons. As MMP-2 and MMP-9 serum concentrations were found to be normally distributed with unequal variances between groups, the Welch analysis of variance (ANOVA) test was used for multiple comparisons between groups. Results are given as mean ± SD or median (IQR) as appropriate unless otherwise indicated. Probability values of < 0.05 were considered statistically significant. Data were analysed using Prism 8.0.2 (GraphPad, San Diego, CA, USA).

## 5. Conclusions

In conclusion, we confirm the beneficial effect of perioperative lidocaine in reducing lung metastasis in a mouse model of breast cancer surgery. When combined with the Src-inhibitor bosutinib, this effect of lidocaine is reversed. Post-mortem serum MMP-2, an enzyme whose expression is increased by Src activation, is significantly reduced in lidocaine-treated animals. These results suggest that reduced MMP-2 may be implicated in the anti-metastatic effect of lidocaine. Whether this is related to an effect on the Src pathway or not remains unclear, and other mechanisms may be involved. Evaluation of the effect of perioperative lidocaine on human cancer recurrence via a prospective, randomized clinical trial is planned [38].
